# Beneficial effect on serum cholesterol levels, but not glycaemic regulation, after replacing SFA with PUFA for 3 d: a randomised crossover trial

**DOI:** 10.1017/S0007114520003402

**Published:** 2021-04-28

**Authors:** Line Gaundal, Mari C. W. Myhrstad, Lena Leder, Marte Gjeitung Byfuglien, Terje Gjøvaag, Ida Rud, Kjetil Retterstøl, Kirsten B. Holven, Stine M. Ulven, Vibeke H. Telle-Hansen

**Affiliations:** 1 Faculty of Health Sciences, Oslo Metropolitan University, 0130 Oslo, Norway; 2 Mills AS, 0558 Oslo, Norway; 3 Nofima (Norwegian Institute of Food, Fisheries and Aquaculture Research), 1433 Ås, Norway; 4 Department of Nutrition, Institute of Basic Medical Sciences, Faculty of Medicine, University of Oslo, 0317 Oslo, Norway; 5 The Lipid Clinic, Department of Endocrinology, Morbid Obesity and Preventive Medicine, Oslo University Hospital, 0424 Oslo, Norway; 6 The Norwegian National Advisory Unit on Familial Hypercholesterolemia, Department of Endocrinology, Morbid Obesity and Preventive Medicine, Oslo University Hospital Rikshospitalet, 0424 Oslo, Norway

**Keywords:** Fatty acids, Glycaemic regulation, Cholesterol, Healthy subjects, Randomised crossover trials

## Abstract

Replacing intake of SFA with PUFA reduces serum cholesterol levels and CVD risk. The effect on glycaemic regulation is, however, less clear. The main objective of the present study was to investigate the short-term effect of replacing dietary SFA with PUFA on glycaemic regulation. Seventeen healthy, normal-weight participants completed a 25-d double-blind, randomised and controlled two-period crossover study. Participants were allocated to either interventions with PUFA products or SFA products (control) in a random order for three consecutive days, separated by a 1·5-week washout period between the intervention periods. Glucose, insulin and TAG were measured before and after an oral glucose tolerance test. In addition, fasting total cholesterol, NEFA and plasma total fatty acid profile were measured before and after the 3-d interventions. Fasting and postprandial glucose, insulin, and TAG levels and fasting levels of NEFA and plasma fatty acid profile did not differ between the groups. However, replacing dietary SFA with PUFA significantly reduced total cholesterol levels by 8 % after 3 d (*P* = 0·002). Replacing dietary SFA with PUFA for only 3 d has beneficial cardio-metabolic effects by reducing cholesterol levels in healthy individuals.

Metabolic diseases such as CVD and type 2 diabetes (T2D) represent major health challenges in the world today^(1)^. People with T2D have 2–4 times higher risk for CVD than non-diabetic individuals of which is the primary cause of death^(2)^. Diet and lifestyle-related factors are major contributors to the development of both CVD and T2D. Improving dietary fat quality, that is exchanging intake of dietary SFA with PUFA, effectively reduces serum cholesterol levels^(3–7)^ and the risk for CVD^(5,8)^. While the SFA lauric acid (12 : 0), myristic acid (14 : 0) and palmitic acid (16 : 0) increase cholesterol levels^(9,10)^, the beneficial cholesterol-lowering effect of dietary fat has in particular been attributed to linoleic acid (LA) (18 : 2*n*-6), the major dietary *n*-6 PUFA found in vegetable oils^(9,11)^. Furthermore, findings from both cohort studies and intervention studies suggest a protective role of LA on the development of T2D^(12–16)^. A recent systematic review and meta-analysis of randomised controlled trials showed that intake of PUFA, mainly LA, favourably influenced risk factors related to T2D development such as glycaemic regulation^(17)^. However, other studies have failed to show any effects^(15,18–23)^. The evidence of fat quality on risk factors of T2D therefore remains limited and elusive, and more studies are needed^(24)^.

Even though the effect of fat quality on metabolic risk factors is well established, the effect has mainly been shown in intervention studies lasting for more than 3 weeks^(5,17)^. Findings from animal and human intervention studies suggest that dietary factors may influence glucose metabolism within a few days^(25–27)^. However, there are currently few studies investigating the short-term effect of changing the dietary fat quality on cardio-metabolic risk factors. The main objective of the present study was therefore to investigate the effect on glycaemic regulation after exchanging intake of SFA with PUFA in a 3-d intervention in healthy individuals.

## Methods

### Study design and subjects

**Fig. 1.**
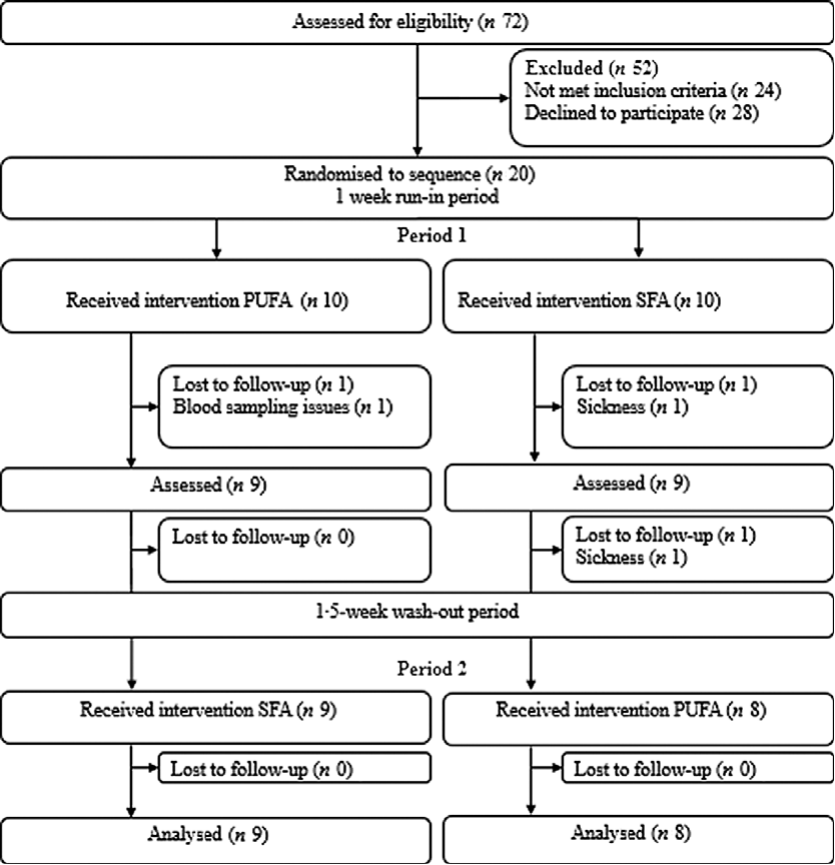
Flow chart of the participants.

**Fig. 2.**
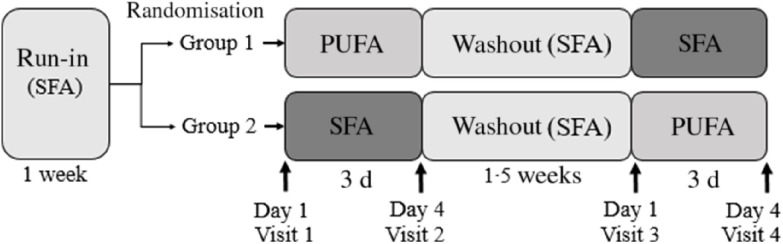
Study design of the double-blind, randomised, controlled crossover study in which seventeen healthy volunteers (group 1: *n* 9; group 2: *n* 8) received daily PUFA products (two muffins and 20 g margarine spread) or SFA products (two muffins and 20 g butter-based spread) for three consecutive days, separated by a 1·5-week washout period. The participants received SFA products in the run-in and washout periods. Fasted blood (glucose, insulin, TAG and total cholesterol) and postprandial blood glucose, insulin and TAG were measured after an oral glucose tolerance test at each visit before and after the 3-d interventions (day 1 and day 4). Body composition was measured fasted at each visit, and physical activity was measured throughout the study period (from run-in to visit 4).

Healthy volunteers were recruited to participate in this double-blind, randomised, controlled crossover study. The study lasted for 25 d and was performed at Oslo Metropolitan University (OsloMet) between April 2018 and January 2019. Twenty participants were randomised and seventeen completed the study. Participants lost during follow-up, and those included in the statistical analyses are outlined in the flow chart (Fig. 1). The participants completed a FFQ (past 12 months) before study start. After a 1-week run-in period, the participants received either PUFA products (margarine and muffins with margarine) or SFA products (control) (butter-based spread and muffins with butter-based spread) in a random order for three consecutive days, followed by a 1·5-week washout period between the intervention periods (Fig. 2). In the run-in and washout periods, the participants were given SFA (control) products. Eligible participants were randomised to a 1:1 allocation ratio to one of two intervention periods: PUFA products or SFA products (control) for three consecutive days, before crossed over to the other intervention period (Fig. 2). A researcher not involved in data collection or first-hand analysis generated the random allocation to treatment sequence with the purpose of allocating a specific number to every volunteer and ensured that information about group allocation remained concealed. When study visits were completed, the researcher provided the codes for unblinding and treatment grouping. Physical activity was measured by a three-axial accelerometer throughout the study period. The participants were instructed to wear the accelerometer each day during the study except from situations where they were in contact with water (i.e. showering, swimming etc.) and during sleep. Clinical assessment and blood sampling were performed before and after both intervention periods (day 1 and day 4). Blood samples were collected after an overnight fast (≥12 h) and after a glucose challenge (oral glucose tolerance test (OGTT)). A case report form was used to assess compliance to the regimen, changes in diet or physical activity level, overall health status, and if the participants reported adverse effects. Participants were instructed to return food containers and any remaining test products at each visit.

Volunteers were recruited from the student mass and employees at OsloMet and through advertisement on Facebook and OsloMet website in 2018. Healthy, normal-weight (BMI between 18·5 and 27 kg/m^2^) adults between 18 and 65 years were included in the study. Participants had to be willing to limit their intake of dietary fats and products rich in *β*-glucan, known to reduce cholesterol levels (oat- and barley-based products) 1 week prior to and during the study. Any use of dietary supplements (e.g. fish oil) or probiotic products (lactic acid bacteria) 4 weeks prior to and during the study was not allowed. Exclusion criteria were fasting blood glucose values ≥6·1 mmol/l, micro C-reactive protein >10 mg/l, chronic diseases (e.g. diabetes, CVD and cancer), intestinal diseases (e.g. inflammatory bowel diseases, celiac disease and irritable bowel disease) and food allergies or intolerances. Further exclusion criteria were antibiotic treatment the previous 3 months and during the study, blood donors the previous 2 months and during the study, pregnancy or lactation, planned weight reduction and/or 5 % weight change the previous 3 months, high alcohol consume (>40 g/d), use of tobacco and hormonal treatment (except oral contraception). The participants were advised to maintain their habitual diet and physical activity level throughout the study, except from the dietary restrictions and inclusion of the study products provided during the study. The study was conducted according to the guidelines laid down in the Declaration of Helsinki, and all procedures involving human subjects were approved by the Regional Committees for Medical and Health Research Ethics (2018/104). Written informed consent was obtained from all subjects. The study was registered at Clinical Trials (https://clinicaltrials.gov/, registration identification number: NCT03658681).

### Study products

In the present study, chocolate muffins and spreads (margarine or butter-based spread (70 % butter and 30 % rapeseed oil), with different fat quality (i.e. either high in PUFA or SFA)) were exchanged with fatty products in the participants regular diet. The total amount of fat given to the participants was based on a study by Vessby *et al.*^(28)^, showing that a beneficial impact of fat quality on insulin sensitivity is only seen in individuals with a fat intake <37 energy % (E%). We therefore calculated the intake of fat to be <37 E%, including the intervention products. The participants were instructed to consume two muffins and a minimum of 20 g spread/d. The study products were to be exchanged for fat-containing products in their regular diet throughout the study. The experimental and control products were similar except from the fat source. The content of SFA in the control products was replaced by sunflower and rapeseed oil, rich in *n*-6 PUFA, in the experimental products (Mills AS). The fatty acid content of the study products is presented in Table 1. During the run-in and washout periods, fat-rich products in the participants’ regular diet were exchanged with the SFA (control) products. No adverse effects were reported during the study periods. The PUFA and SFA (control) muffins were prepared at different days and stored at −20°C at OsloMet. The margarine and butter-based spread were packed in neutral packaging by Mills AS and sent to OsloMet in parallel with the recruitment and kept at 4°C at OsloMet for maximum 4 weeks. The participants received the study products at each visit. A routine food analysis laboratory (Eurofins Food & Feed Testing Norway AS) analysed the fatty acid content and composition of the study products at the end of the study (Table 1).

**Table 1.** Fatty acid composition of the study products

	SFA			PUFA		
	Butter-based spread* (g/100 g)	Muffin (g/100 g)	Total products† (g/d)	Margarine (g/100 g)	Muffin (g/100 g)	Total products† (g/d)
Total fat	85·3	31·2	79·5	59·9	28·9	69·8
Total SFA	31·8	11·8	29·9	11·6	<0·03	2·4
Total MUFA	36·0	13·1	33·4	26·1	12·9	30·9
Total PUFA	10·9	4·0	10·2	19·4	11·3	26·4
Sum *n-*6 fatty acids	7·5	2·9	7·3	17·4	10·1	23·7
Sum *n-*3 fatty acids	3·3	1·1	2·9	2·0	1·2	2·7
Lauric acid (12 : 0)	1·3	0·5	1·3	1·4	<0·03	0·3
Myristic acid (14 : 0)	4·7	1·6	4·1	0·6	<0·03	0·2
Palmitic acid (16 : 0)	16·2	6·1	15·4	3·1	1·8	4·2
Stearic acid (18 : 0)	6·4	2·4	6·1	5·7	1·1	3·3
Oleic acid (18 : 1)	34·4	12·5	31·9	25·5	12·6	30·3
Linoleic acid (18 : 2*n-*6)	7·4	2·9	7·3	17·4	10·1	23·6
*α*-Linolenic acid (18 : 3*n-*3)	3·3	1·1	2·9	2·0	1·2	2·7

* The butter-based spread contained 70 % butter and 30 % rapeseed oil.

† The participants were to eat 20 g butter-based spread or margarine and 200 g muffin per d containing butter-based spread or margarine, respectively.

### Blinding

The food containers with margarine and butter-based spread and the chocolate muffins had similar appearance. The participants were randomly allocated to begin with either the SFA or the PUFA intervention. The randomisation list consisted of the ID numbers, and only personnel packing the study products had access to the list and knew who were allocated to which intervention. The study products were stored in paper bags at OsloMet and labelled with the ID numbers and handed out to the participants at each visit. Hence, the present study was double blind, as neither the participants nor the personnel handing out the products knew which fat quality the products consisted.

### Oral glucose tolerance test

A standard OGTT was performed at each visit. A quantity of 82 g of glucose (d(+)-glucose monohydrate), equal to 75 g glucose, was dissolved in 100 ml water and stored in a refrigerator for a maximum of 2 weeks. The participants were instructed to consume the OGTT within 10 min and to remain seated between the measurements following the OGTT. Finger-prick capillary blood samples for glucose measurements were taken before and 15, 30, 60, 90, 120, 150 and 180 min after the OGTT. Venous blood samples were taken before and 30, 60 and 120 min after the OGTT.

### Blood sampling and laboratory analyses

The participants were instructed to refrain from alcohol consumption and excessive physical activity the day before blood sampling. Blood samples were collected after an overnight fast (≥12 h) at each visit. Glucose was measured with a HemoCue Glucose 201 Analyser and Micro cuvettes following a standardised procedure^(29)^. The HemoCue 201 Micro cuvettes were stored in a refrigerator (4°C) and taken out in room temperature 30 min prior to blood sampling. Furthermore, TAG, insulin and total cholesterol were analysed in serum. Serum was obtained from 8·5 ml serum gel tubes and turned 6–10 times before spin down after 30 min (1300–1500 ***g***, 15 min), and kept in a refrigerator (4°C) before it was sent to a routine laboratory (Fürst Medical Laboratory) within 24 h.

### Fatty acid analysis

NEFA were measured using an enzymatic colorimetric assay with acyl-CoA oxidase and MEHA as a colour reagent. Total plasma (EDTA) fatty acid profile was measured with GC flame ionising detection. Internal standard (triheptadecanoin) was added, and samples were methylated with 3 m HCl in methanol. Fatty acid methyl esters were extracted with hexane, and then samples were neutralised with 3 m KOH in water. After mixing and centrifuging, the hexane phase was injected into the GC flame ionising detection. Analysis was performed on a 7890A GC with a split/splitless injector, a 7683B automatic liquid sampler and flame ionisation detection (Agilent Technologies). Separations were performed on a SP-2380 (30 m × 0·25 mm internal diameter × 0·25 µm film thickness) column from Supelco. The concentration of the individual fatty acids was measured as µg fatty acid/ml plasma and presented as percentage of total fatty acids. Both analyses were performed at a commercial laboratory (Vitas Analytical Service).

### Anthropometry

Body weight and composition were measured after an overnight fast at each visit using a Tanita scale (BC-418 Segmental Body Composition Analyser). Any metal (i.e. watch, jewellery, belt, etc.), shoes and socks were removed before the measurement. To compensate for clothing, 1 kg was subtracted from the body weight. Height was measured by a wall-mounted stadiometer.

### Physical activity

A three-axial accelerometer (ActiGraph GTX3, Actigraph Corporation) was used to objectively measure physical activity. Participants were asked to wear the accelerometer for a total of 25 d, that is for 1 week during the run-in period, 3 d during the first intervention period, 1·5 week during the washout period and 3 d during the second intervention period (Fig. 2). The accelerometer was worn at the left hip throughout waking hours, and the participants were instructed to remove the device only for showering and during sleep. Following the 25 d data collection period, the data were downloaded using the ActiLife 6 software (ActiGraph Corporation). Wear and non-wear-time classification was then performed according to Choi *et al.*^(30)^ and wear-time validated data were summarised into daily average counts (counts per minute; cpm). For each participant, and for all the different time periods (i.e. the interventions, run-in and washout periods), the same three weekdays (no weekend days) were used. Summary averages for the 3-d activity periods were computed only for participants that wore the monitor ≥10 h/d during all three measuring days^(31)^. Participants who wore the monitor for <10 h/d were not included in the analysis. Hence, for the analysis of physical activity, the number of participants varies from fourteen to seventeen (online Supplementary Table S2). Furthermore, Freedson three-axis vector magnitude accelerometer count threshold values^(32)^ were used to characterise accelerometer counts as percentage time spent in light (0–2690 cpm), moderate (2691–6166 cpm), vigorous (6167–9642 cpm) and very vigorous intensity physical activity (≥9643 cpm).

**Table 2.** Baseline characteristics of the participants (Medians and 25th–75th percentiles)

	Median	25th–75th percentiles
Male/female (*n*)	6/11	
Age (years)	28·0	25·0–46·0
BMI (kg/m^2^)	22·8	22·0–25·0
HbA1c (%)*	5·2	5·0–5·4
HbA1c (mmol/mol)*	33·0	31·2–35·5
Glucose (mmol/l)	5·1	4·9–5·4
Insulin (pmol/l)	51·0	31·0–60·0
TAG (mmol/l)	0·9	0·6–1·4
Total cholesterol (mmol/l)	4·9	4·4–5·4
mCRP (mg/l)	0·9	0·3–1·4
Systolic blood pressure (mmHg)*	123	113–136
Diastolic blood pressure (mmHg)*	71	66–75
Body fat (%)	24·4	12·5–33·9
FFM (kg)	47·8	45·3–69·3

mCRP, micro C-reactive protein; FFM, fat-free mass.

* HbA1c, systolic and diastolic blood pressure measured at screening. Variables are measured fasted.

### Statistical analysis

The primary endpoint of the present study was changes in metabolic regulation, measured as blood glucose and insulin response fasting and during an OGTT. Secondary endpoints were homoeostasis model assessment of insulin resistance, Matsuda index and blood lipids (TAG and total cholesterol). Sample size was based on the existing literature on fat and glycaemic regulation. Several of these studies have however compared the effect of SFA with carbohydrates or PUFA to other unsaturated fatty acids or have used other methods to measure glycaemic response, and we were unable to perform power calculations^(16,17,33)^. We therefore aimed to include 20–30 participants in this study. Due to sample size, data were analysed with non-parametric tests and are presented as medians and 25–75th percentiles. The Wilcoxon signed-rank test was used to assess differences between interventions (SFA *v.* PUFA), and within interventions (day 1 *v*. day 4), and potential changes in body weight and composition from baseline to the end of the study. The blood glucose, insulin and TAG AUC were calculated for each participant using the trapezoidal rule (A = (*y*_1_ + *y*_2_) × (*x*_2_ − *x*_1_)/2). The incremental AUC was calculated for each participant by subtracting the fasting value (0 min) at each visit from the corresponding values after the OGTT, and thereafter using the trapezoidal rule. The homoeostasis model assessment of insulin resistance^(34)^ and Matsuda index^(35)^ were calculated for each participant to assess insulin resistance and insulin sensitivity, respectively. One-way ANOVA was used to analyse potential differences in 3-d physical activity patterns during the run-in, washout and intervention periods and performed blinded with regard to the nutritional content (SFA *v.* PUFA) in the intervention periods. *P* < 0·05 was regarded as statistically significant. For any missing values, the mean value for a missing time point was calculated based on the same time point during the other visits for the particular participant. All statistical analyses were performed in IBM SPSS statistics (version 25) after processing the data in Microsoft Excel 2016 for Windows (16.0.4849.1000). Figures were designed using GraphPad Prism 8 for Windows (version 8.0.0.).

## Results

Seventeen participants (six males, eleven females) completed this double-blind, randomised crossover study, and the baseline characteristics of the participants are shown in Table 2. The participants were normal-weight (median BMI of 22·8 kg/m^2^, 25th–75th percentiles: 22·0–25·0) adults (median age of 28 years, 25th–75th percentiles: 25·0–46·0), with fasting blood glucose, total cholesterol and TAG levels within the normal range. Information about the participants’ background diet was collected by a FFQ and is shown in online Supplementary Table S1.

**Fig. 3.**
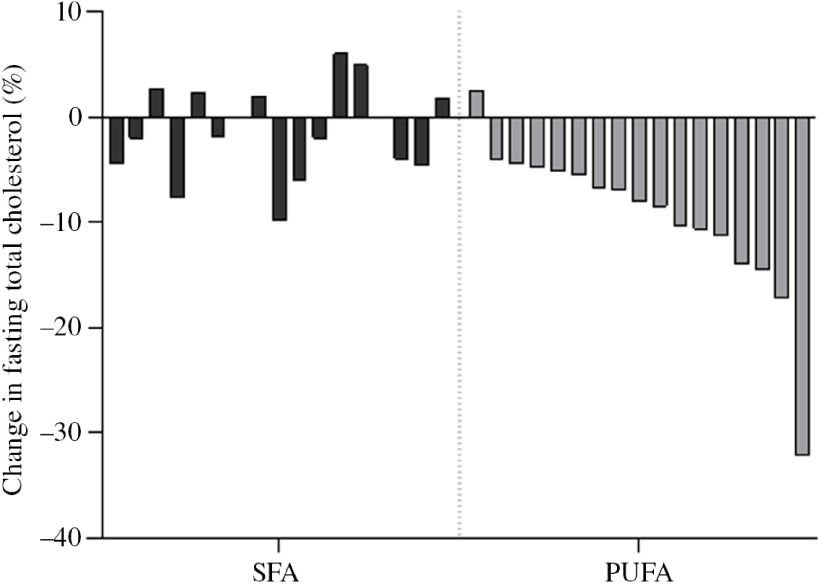
Individual changes in fasting total cholesterol. Each bar represent individual percentage change in fasting total cholesterol level after 3-d intervention with SFA and PUFA.

In order to investigate the effect of PUFA and SFA products on glycaemic regulation, glucose and insulin levels were measured before and after an OGTT. The results indicate that intake of study products rich in PUFA for 3 d did not change glycaemic response compared with intake of SFA products (Table 3). The blood glucose level at 15 min after the OGTT significantly decreased after the SFA intervention (*P* = 0·038), but did not significantly differ from the PUFA intervention (Table 4). Insulin sensitivity and resistance measured as the Matsuda index and homoeostasis model assessment of insulin resistance, respectively, were not significantly changed after intake of PUFA products compared with SFA products. However, insulin sensitivity measured by the Matsuda index slightly increased after the SFA intervention (*P* = 0·049) (Table 3). Fasting total cholesterol was measured before and after the 3-d interventions with SFA and PUFA. Intake of PUFA products significantly reduced the total cholesterol level compared with intake of SFA (*P* = 0·002) (Table 5). The median total cholesterol levels decreased with 0·4 mmol/l after PUFA intervention and 0·1 mmol/l after the SFA intervention. The 0·4 mmol/l change within the PUFA intervention corresponded to an 8 % reduction. Interestingly, a significant reduction in total cholesterol was evident in all but one participant (sixteen out of seventeen) after intake of PUFA products. These individual changes ranged from −4 to −32 %, whereas one participant had a 2 % increase (Fig. 3).

**Table 3.** Effects of SFA and PUFA intake on glycaemic regulation (Medians and 25th–75th percentiles)

	SFA					PUFA					
	Day 1		Day 4		*P* †	Day 1		Day 4		*P* ‡	*P* §
	Median	25th–75th percentiles	Median	25th–75th percentiles		Median	25th–75th percentiles	Median	25th–75th percentiles		
Fasting glucose (mmol/l)	5·1	4·9–5·6	5·2	4·8–5·3	0·245	5·3	5·0–5·4	5·2	5·1–5·5	0·263	0·120
Glucose AUC	1076·3	1023·0–1153·0	1063·5	977·3–1149·0	0·381	1080·8	1020·8–1179·0	1074·0	1012·5–1133·3	0·368	0·906
Glucose iAUC	164·3	105·0–232·5	163·5	117·8–231·8	1·000	117·0	108·8–174·8	120·0	84·0–166·5	0·227	0·210
Fasting insulin (pmol/l)	36	32–51	34	27–43	0·469	31	28–68	36	25–46	0·107	0·532
Insulin AUC	21 735	20 625–28 155	19 890	17 385–28 905	0·344	26 280	20 550–38 250	25 755	14 835–34 245	0·332	0·906
Insulin iAUC	17 145	15 615–22 485	15 840	13 185–23 430	0·586	22 455	16 470–26 895	20 715	12 315–24 600	0·381	0·868
Matsuda index||	19·8	18·1–24·0	21·7	18·5–33·7	0·049*	23·4	12·3–24·5	20·9	15·1–33·4	0·193	0·554
HOMA-IR¶	7·8	6·7–12·7	7·3	5·4–10·1	0·332	7·1	6·5–15·9	8·5	5·7–11·2	0·068	0·554

iAUC, incremental AUC; HOMA-IR, homoeostatic measure of insulin resistance; OGTT, oral glucose tolerance test.

* Significance is defined as *P* < 0·05.

† Day 1 *v*. day 4 within SFA intervention; Wilcoxon signed-rank test.

‡ Day 1 *v*. day 4 within PUFA intervention; Wilcoxon signed-rank test.

§ Comparing change from day 1 to day 4 between the PUFA and SFA intervention; Wilcoxon signed-rank test.

|| Matsuda index = 10 000/√((fasting glucose × fasting insulin) × (glucose and insulin during OGTT (0–120 min))).

¶ HOMA-IR = (fasting insulin × fasting glucose)/22·5.

**Table 4.** Effects of SFA and PUFA intake on glycaemic regulation (Medians and 25th–75th percentiles)

	SFA					PUFA					
	Day 1		Day 4			Day 1		Day 4			
	Median	25th–75th percentiles	Median	25th–75th percentiles	*P* †	Median	25th–75th percentiles	Median	25th–75th percentiles	*P* ‡	*P* §
Fasting glucose (mmol/l)	5·1	4·9–5·6	5·2	4·8–5·3	0·245	5·3	5·0–5·4	5·2	5·1–5·5	0·263	0·120
Glucose 15 min (mmol/l)	7·2	6·9–7·5	6·6	6·2–7·3	0·038*	7·4	6·5–7·9	7·4	7·1–7·9	0·703	0·057
Glucose 30 min (mmol/l)	8·1	7·7–9·0	8·6	7·6–8·8	0·605	8·3	7·3–9·5	8·4	7·7–9·1	0·679	0·906
Glucose 60 min (mmol/l)	6·8	6·0–7·7	7·0	6·0–7·5	0·776	6·9	5·9–7·4	6·1	5·9–7·2	0·129	0·236
Glucose 90 min (mmol/l)	6·0	5·4–6·7	6·1	5·5–6·4	0·669	5·8	5·3–6·5	6·0	5·6–6·5	0·569	0·313
Glucose 120 min (mmol/l)	5·8	4·9–7·1	5·2	4·6–6·6	0·604	5·9	5·1–6·4	5·7	5·2–5·9	0·586	0·918
Glucose 150 min (mmol/l)	5·2	4·3–6·2	5·1	4·1–6·3	0·518	5·0	3·9–5·7	4·8	3·7–5·4	0·276	0·602
Glucose 180 min (mmol/l)	4·4	4·0–4·7	4·4	3·8–4·6	0·443	4·3	4·0–4·7	4·3	4·0–4·8	0·954	0·622
Fasting insulin (pmol/l)	36	32–51	34	27–43	0·469	31	28–68	36	25–46	0·107	0·532
Insulin 30 min (pmol/l)	328	251–462	377	207–476	0·492	352	280–508	432	182–570	0·381	0·653
Insulin 60 min (pmol/l)	189	157–258	200	116–239	0·653	245	135–360	170	201–199	0·055	0·309
Insulin 120 min (pmol/l)	100	81–153	85	44–141	0·163	90	51–227	89	58–139	0·943	0·163

* Significance is defined as *P* < 0·05.

† Day 1 *v*. day 4 within SFA intervention; Wilcoxon signed-rank test.

‡ Day 1 *v*. day 4 within PUFA intervention; Wilcoxon signed-rank test.

§ Comparing change from day 1 to day 4 between the PUFA and SFA intervention; Wilcoxon signed-rank test.

**Table 5.** Effects of SFA and PUFA intake on serum lipids (Medians and 25th–75th percentiles)

	SFA					PUFA					
	Day 1		Day 4		*P* †	Day 1		Day 4		*P* ‡	*P* §
	Median	25th–75th percentiles	Median	25th–75th percentiles		Median	25th–75th percentiles	Median	25th–75th percentiles		
Total cholesterol (mmol/l)	5·0	4·4–5·1	4·8	4·3–5·3	0·274	5·0	4·7–5·7	4·6	4·2–4·9	<0·001*	0·002*
Fasting TAG (mmol/l)	0·8	0·6–1·2	0·7	0·5–1·1	0·246	0·9	0·8–1·2	0·8	0·5–0·9	0·002*	0·065
TAG AUC	78·2	71·0–125·9	71·1	59·0–135·2	0·381*	97·8	86·7–129·6	83·3	68·6–120·0	0·006*	0·309
TAG iAUC	−8·9	−14·0 to −5·6	−8·6	−11·7 to −4·2	0·423	−10·8	−15·5 to −1·5	-6·0	−12·8 to 4·0	0·185	0·421
NEFA (mm)	0·2	0·2–0·3	0·2	0·1–0·4	0·552	0·2	0·1–0·3	0·2	0·2–0·3	0·356	0·698

iAUC, incremental AUC.

* Significance is defined as *P* < 0·05.

† Day 1 *v*. day 4 within SFA intervention; Wilcoxon signed-rank test.

‡ Day 1 *v*. day 4 within PUFA intervention; Wilcoxon signed-rank test.

§ Comparing change from day 1 to day 4 between the PUFA and SFA intervention; Wilcoxon signed-rank test.

Fasting and postprandial TAG response after the OGTT were not significantly different after intake of PUFA compared with SFA products (Table 5). However, intake of PUFA products significantly decreased fasting TAG with 11·1 % (*P* = 0·002) from the baseline value, and the TAG AUC during OGTT with 14·8 % (*P* = 0·006).

No changes in the concentration of fasting NEFA were observed after intake of PUFA compared with SFA products (Table 5). Intake of PUFA products for 3 d did not significantly alter the plasma fatty acid profile compared with intake of SFA products (Table 6). However, only within the PUFA intervention did the plasma concentration of the SFA pentadecanoic acid (15 : 0) and palmitic acid (16 : 0) decrease (*P* = 0·030, *P* = 0·039, respectively), whereas the level of stearic acid (18 : 0) increased (*P* = 0·031). Furthermore, the level of arachidonic acid (20 : 4*n*-6) increased (*P* = 0·034), and *α*-linolenic acid (18 : 3*n*-3) decreased (*P* = 0·029) only within the PUFA intervention. The level of LA increased after both the SFA intervention (*P* = 0·011) and PUFA intervention (*P* = 0·013), and no significant difference was observed between the groups (Table 6).

**Table 6.** Effects of SFA and PUFA intake on plasma fatty acids (weight-% of fatty acid methyl esters) (Medians and 25th–75th percentiles)

	SFA					PUFA					
	Day 1		Day 4		*P* †	Day 1		Day 4		*P* ‡	*P* §
	Median	25th–75th percentiles	Median	25th–75th percentiles		Median	25th–75th percentiles	Median	25th–75th percentiles		
Total SFA	29·7	29·4–30·7	29·6	29·3–30·3	0·758	29·9	29·3–30·6	29·4	28·6–30·3	0·130	0·309
12 : 0	0·07	0·05–0·09	0·06	0·05–0·08	0·399	0·06	0·05–0·09	0·06	0·05–0·09	0·526	0·705
14 : 0	0·86	0·76–0·97	0·76	0·67–0·99	0·148	0·80	0·70–1·00	0·72	0·59–0·95	0·052	0·815
15 : 0	0·23	0·20–0·24	0·22	0·19–0·25	0·979	0·24	0·20–0·26|	0·20	0·18–0·23	0·030*	0·102
16 : 0	20·4	19·8–20·9	20·0	19·3–20·7	0·309	20·1	19·7–20·9	19·7	18·8–20·1	0·039*	0·097
18 : 0	6·8	6·2–7·1	6·9	6·4–7·1	0·705	6·8	6·3–6·9	7·1	6·5–7·3	0·031*	0·255
Total MUFA	22·9	21·4–24·0	21·2	20·3–22·3	0·007*	21·9	21·2–24·1	20·7	19·5–22·6	0·049*	0·887
16 : 1*c*9	1·50	1·19–1·85	1·31	1·13–1·73	0·102	1·46	1·24–1·85	1·18	0·94–1·42	0·052	0·351
18 : 1*c*9	19·5	18·2–21·3	18·6	17·7–18·8	0·006*	19·1	18·4–20·5	18·4	17·2–19·5	0·028*	0·925
18 : 1*c*11	1·39	1·32–1·53	1·44	1·37–1·57	0·266	1·43	1·33–1·59	1·50	1·29–1·58	0·849	0·427
Total PUFA	40·7	40·0–42·1	43·5	40·0–42·1	0·007*	42·0	38·7–43·1	43·6	42·2–44·3	0·025*	0·463
*n*-6 PUFA	36·0	34·1–37·7	38·1	36·2–38·8	0·003*	37·0	33·2–38·6	39·5	36·1–40·7	0·013*	0·407
18 : 2*n*-6	28·0	26·6–30·1	30·1	27·4–31·9	0·011*	27·9	25·4–30·7	30·0	27·7–32·7	0·013*	0·517
18 : 3*n*-6	0·31	0·24–0·36	0·24	0·16–0·29	0·155	0·31	0·24–0·40	0·29	0·21–0·35	0·148	0·744
20 : 3*n*-6	1·20	1·04–1·32	1·13	0·97–1·36	0·776	1·26	1·03–1·42	1·24	1·00–1·43	0·179	0·275
20 : 4*n*-6	5·9	4·7–6·7	5·9	5·1–6·8	0·492	6·1	5·5–7·4	6·5	5·6–7·7	0·034*	0·142
*n*-3 PUFA	4·9	4·6–5·7	5·1	4·7–5·5	0·758	4·9	4·5–5·4	4·4	4·0–5·0	0·065	0·344
18 : 3*n*-3	0·94	0·90–1·07	0·89	0·85–1·08	0·453	0·97	0·88–1·07	0·82	0·72–1·04	0·029*	0·283
20 : 5*n*-3	1·00	0·82–1·42	0·95	0·77–1·34	0·149	1·00	0·83–1·21	0·87	0·72–0·94	0·113	0·977
22 : 5*n*-3	0·49	0·44–0·56	0·47	0·45–0·50	0·194	0·48	0·44–0·56	0·48	0·43–0·54	0·392	0·388
22 : 6*n*-3	2·45	2·16–2·88	2·66	2·39–2·96	0·142	2·37	1·99–2·69	2·36	2·01–2·75	0·368	0·355

* Significance is defined as *P* < 0·05.

† Day 1 *v*. day 4 within SFA intervention; Wilcoxon signed-rank test.

‡ Day 1 *v*. day 4 within PUFA intervention; Wilcoxon signed-rank test.

§ Comparing change from day 1 to day 4 between the PUFA and SFA intervention; Wilcoxon signed-rank test.

Physical activity level was monitored throughout the study using a three-axial accelerometer. The level of light, moderate, vigorous and very vigorous activity remained stable throughout the study (online Supplementary Table S3). Furthermore, BMI and body composition (fat percentage and fat-free mass) remained stable throughout the study (data not shown).

## Discussion

The present study clearly shows that replacing intake of products high in SFA with products high in PUFA for only 3 d reduced serum total cholesterol levels in healthy, young adults, whereas glycaemic response remained unchanged.

This is to our knowledge the first study to show a reduced total cholesterol level of 0·4 mmol/l, corresponding to an 8 % decrease, after intake of products high in PUFA for only 3 d.

Despite the rapid and clear effect on total cholesterol levels after replacing intake of products high in SFA with PUFA, we did not observe any significant effects on glycaemic regulation. The participants in the present study were healthy, physically active, normal-weight adults, and their body weight remained stable throughout the study. Findings from observational studies^(36)^ and controlled trials^(16,37,38)^ demonstrate that intake of PUFA was associated with improved insulin sensitivity only among obese participants. In healthy participants, excess intake of either SFA or PUFA for 7 weeks modestly induced weight gain and subsequently increased insulin levels and insulin resistance irrespective of fat quality^(39)^. Hence, these findings suggest that fat quality may be less potent in regulating glucose metabolism in physically active, healthy, weight-stable individuals. The total fat intake in the present study was estimated to be maximum 37 % of total energy intake. This was based on a study by Vessby *et al*. showing a beneficial impact of fat quality on insulin sensitivity when total fat intake was <37 %^(28)^. The Nordic Nutrition Recommendations includes 25–40 % of total energy from fat^(40)^. Hence, the amount of fat in the present study is in line with the recommendation of total fat in the diet and could therefore be feasible for long term. However, the present study was not a fully controlled dietary intervention study, and even though the participants were to exchange included fat-rich products in their habitual diet with the SFA- and PUFA-rich study products, intake of SFA and PUFA from other food sources may have occurred. Hence, the participants may have consumed a larger amount of fat (exceeding 37 % of total energy intake) that might explain the lack of effect on glycaemic response.

The present data show that serum total cholesterol reflects dietary habits for as short as 3 d prior to the blood test. Studies have shown that replacing dietary SFA with PUFA reduces total cholesterol levels with about 0·6 mmol/l, or 9–10 %^(3)^, and a systematic review and meta-analysis of randomised controlled trials showed that exchanging intake of SFA with PUFA reduced total cholesterol levels in the same range (about 0·76 mmol/l)^(6)^. This is line with the effect achieved in the present study and indicates that changes in the dietary intake of fat quality alter cholesterol metabolism.

The reduced total cholesterol level in the present study is in line with findings from intervention studies of longer duration^(3,6,18)^. Most studies investigating changes in cholesterol levels related to diet lasts for several weeks to months and years^(3,5,10,18,41)^. In a recent study by Vedel-Krogh *et al.*, total cholesterol was increased in the first week of January, immediately after the Christmas holidays and 89 % of the subjects had total cholesterol levels above the recommended 5 mmol/l. Furthermore, the total cholesterol level was 15 % higher in subjects measured in December–January compared with those measured in May–June^(42)^. Taken together, this indicates that fluctuations in cholesterol levels throughout the year may coincide with short-term changes in diet. Hence, future studies should investigate day-by-day effects of diet on cholesterol levels.

In the present study, the participants were healthy, young adults with total cholesterol levels within the normal range. Despite of this, intake of PUFA significantly reduced total cholesterol levels and this was observed in sixteen out of seventeen participants, ranging from 4 to 32 % reduction. Similarly, Retterstøl *et al.* observed large individual differences in total cholesterol in young, normal-weight and healthy individuals after a dietary intervention^(43)^. They investigated the effect of a low-carb high-fat diet for 3 weeks and found that total cholesterol increased 10–70 %^(43)^. The concept of the lifelong cholesterol burden of an individual as a main determinant for defining the risk of CVD has been suggested^(44,45)^. Therefore, exchanging intake of SFA with PUFA clearly has an impact on future CVD risk by reducing the total cholesterol level also in young, healthy adults with cholesterol levels within the normal range.

In order to improve fat quality in the present study, the *n*-6 PUFA LA replaced most of the SFA content in the food items provided in the study. LA is an essential *n*-6 fatty acid, and there is evidence that *n*-6 PUFA is protective for CVD^(5,6,8,11)^. An intake of LA of at least 8 % of total energy is associated with lower mortality^(46)^ and reduced CVD risk independent of other dietary factors^(11)^. In a Cochrane report from 2018, the total cholesterol was reduced by 0·33 mmol/l after intake of *n*-6 PUFA in randomised controlled trials lasting from 1 to 12 years^(41)^. Our results are in line with these findings showing a beneficial effect on total cholesterol after replacing SFA with PUFA, in particular LA, in the diet.

In the present study, we only measured total cholesterol and hence we do not know which of the lipoproteins that are affected. It is well known that fatty acids, and in particular PUFA, are ligands for nuclear receptors involved in lipid metabolism, including the PPAR. Activation of PPAR by PUFA will increase the transcription of genes involved in lipid metabolism including fat oxidation^(47,48)^. Increased hepatic fatty acid oxidation by activation of PPAR will reduce the fatty acids available for esterification into TAG, cholesterol esters and phospholipids and thereby a reduced production of VLDL. This may explain the reduced total cholesterol level in plasma observed in the present study. Furthermore, PUFA also have the potential to reduce the expression of genes involved in fatty acid and cholesterol synthesis via the transcription factor sterol regulatory element binding protein 1. Sterol regulatory element binding protein 1 activates the expression of several genes involved in the synthesis and uptake of cholesterol, fatty acids, TAG and phospholipids^(48)^. Interestingly, findings from postprandial studies indicate that fat quality may have a rapid impact on transcriptional regulation. A postprandial crossover study conducted in healthy individuals has shown that intake of PUFA affected expression of genes related to cholesterol metabolism differently than SFA after only 6 h^(49)^. Furthermore, another postprandial study in subjects with and without familial hypercholesterolaemia showed that intake of SFA compared with *n*-6 PUFA induced changes in expression of genes related to cholesterol metabolism after 4–6 h^(50)^. These rapid transcriptional effects may potentially explain the beneficial effect on serum cholesterol after intake of PUFA compared with SFA. We cannot exclude that other mechanistically explanations such as post-translational modifications caused the observed effect on cholesterol metabolism. Future studies investigating the short-term effect of fat quality on cholesterol metabolism are warranted and should include a mechanistic approach, such as analyses on gene expression.

The difference in the content of LA between the intervention products was not reflected in higher serum LA levels after the PUFA intervention compared with SFA. Previous studies have shown that increased intake of LA will be reflected in plasma and will reach a steady state after approximately 2 weeks^(51)^. LA from the diet will reach the liver, where it may be esterified or oxidised. In addition, LA has the potential to be further elongated and desaturated to very long-chain PUFA. Thus, short-term effect on changes in LA intake might therefore not necessarily be reflected in the plasma LA levels. Furthermore, the SFA-containing food items in the present study originated from dairy fat, using a commercially available butter-based spread. Here, we show that intake of study products high in PUFA significantly reduced the level of pentadecanoic acid (15 : 0), a biomarker of dairy fat intake^(52)^, suggesting a decreased intake of dairy SFA. Taken together, it is therefore reasonable to assume that the improved fat quality most likely caused the observed effects in the present study.

Our findings suggest that intake of PUFA may elicit cardio-protective effects through both affecting cholesterol and TAG metabolism. Increased postprandial TAG levels have been associated with increased risk for CHD^(53)^ and may predict cardiovascular events^(54)^. As serum TAG level is an independent risk factor for CVD^(55,56)^, our findings suggest that intake of PUFA may elicit cardio-protective effects through both affecting cholesterol and TAG metabolism.

The small sample size in the present study is a limitation especially related to the primary endpoint, although sufficient to demonstrate significant changes in lipid metabolism. The effect was observed in young, healthy, normal-weight participants and cannot be generalised to the population as a whole. Nevertheless, the present study has several strengths. First, the crossover design allowed each participant to act as his/her own control, avoiding any biases such as biological differences between participants. Second, they were randomly allocated to begin with either the SFA or the PUFA intervention, thus avoiding any bias of period effect during the study. Third, the participants consumed products rich in SFA both in the run-in and washout periods; hence, the observed effects can be attributed to the replacement of SFA with PUFA. The randomisation and the 1·5-week washout period thus minimised possible effects of treatment order and carryover effect. Fourth, they were instructed to refrain from products containing *β*-glucan, known to reduce cholesterol levels^(57,58)^, thus suggesting that improved dietary fat quality elicited the observed effects. Finally, monitoring physical activity throughout the study period strengthens the findings in the present study. The participants had a stable weight and physical activity level throughout the study, suggesting that our findings can be attributed to the changes induced by diet, and not by changes in weight or physical activity level.

In conclusion, we showed that by exchanging SFA with PUFA in the diet for only 3 d, cholesterol levels were reduced by 8 % in healthy, weight-stable young adults. Small changes in cholesterol levels implemented early are associated with reduced risk of CVD later in life.

## Supplementary material

For supplementary material accompanying this paper visit https://doi.org/10.1017/S0007114520003402. click here to view supplementary material
