# Supplementary material for: Beneficial effect on serum cholesterol levels, but not glycaemic regulation, after replacing SFA with PUFA for 3 d: a randomised crossover trial
Source: Br J Nutr. 2020 Sep 2;125(8):915–25. doi: 10.1017/S0007114520003402 (PMC7944393; doi:10.1017/S0007114520003402)
Supplement: Supplementary file 1 [file S0007114520003402sup001.docx]

**Supplemental Table 1.** Macronutrient intake assessed by food frequency questionnaire (FFQ).

|  | Median (g) | 25th - 75th percentiles | Median (E %) | 25th - 75th percentiles |
| --- | --- | --- | --- | --- |
| KJ | 9980.0 | 8226.0 - 13887.0 |  |  |
| Kcal | 2385.3 | 1966.1 - 3319.1 |  |  |
| Protein | 100.3 | 79.7 - 137.0 | 15.7 | 14.0 - 16.5 |
| Fat | 110.4 | 72.1 - 121.0 | 34.8 | 31.4 - 36.5 |
| SFA | 33.2 | 24.2 - 44.3 | 11.6 | 11.1 - 12.9 |
| MUFA | 41.4 | 31.2 - 44.4 | 14.0 | 11.6 - 15.7 |
| PUFA | 20.0 | 14.8 - 23.9 | 6.5 | 5.3 - 8.4 |
| n-6 | 14.1 | 11.2 - 17.1 | 4.5 | 3.9 - 5.9 |
| n-3 | 4.2 | 3.2 - 6.0 | 1.4 | 0.9 - 1.9 |
| Carbohydrates | 254.0 | 233.2 - 345.0 | 43.8 | 39.3 - 47.3 |
| Fiber | 39.6 | 31.8 - 51.7 |  |  |

Abbreviations: FFQ, food frequency questionnaire; MUFA, monounsaturated fatty acid; n-3, omega-3; n-6, omega-6;

PUFA, polyunsaturated fatty acid; SFA, saturated fatty acid.

**Supplemental Table 2.** Percent time of physical activity in specific intensity levels^1^

|  | Light activity^2^ | |  | Moderate activity^3^ | |  | Vigorous activity^4^ | |  | Very vigorous activity^4^ | |  | MVPA^2^ | |
| --- | --- | --- | --- | --- | --- | --- | --- | --- | --- | --- | --- | --- | --- | --- |
|  | Mean | SD |  | Mean | SD |  | Mean | SD |  | Mean | SD |  | Mean | SD |
| Run-in period | 88.99 | 3.64 |  | 9.23 | 2.93 |  | 1.54 | 1.53 |  | 0.24 | 0.48 |  | 11.05 | 3.73 |
| SFA-intervention | 89.23 | 2.30 |  | 9.15 | 2.54 |  | 1.40 | 1.11 |  | 0.20 | 0.34 |  | 10.76 | 2.30 |
| Wash-out period | 88.61 | 3.95 |  | 9.32 | 3.29 |  | 1.92 | 1.91 |  | 0.17 | 0.31 |  | 11.40 | 3.99 |
| PUFA-intervention | 90.08 | 3.05 |  | 8.47 | 2.16 |  | 1.35 | 1.32 |  | 0.09 | 0.18 |  | 9.91 | 3.05 |
| *P^5^* | 0.66 |  |  | 0.83 |  |  | 0.69 |  |  | 0.69 |  |  | 0.65 |  |

MVPA, moderate and vigorous physical activity; PUFA, Polyunsaturated fatty acids; SFA, Saturated fatty acids.

^1^The data are given as mean and SD values in three-day periods for the SFA and PUFA-interventions, run-in and wash-out period.
^2^n=15
^3^n=17
^4^n=14
^5^Between group comparison; One way ANOVA
